# Multimodal evaluation of an Italian family with a hereditary spastic paraplegia and *POLR3A* mutations

**DOI:** 10.1002/acn3.51221

**Published:** 2020-10-21

**Authors:** Lucia Ruggiero, Aniello Iovino, Raffaele Dubbioso, Sirio Cocozza, Rosanna Trovato, Francesco Aruta, Giuseppe Pontillo, Melissa Barghigiani, Arturo Brunetti, Filippo Maria Santorelli, Fiore Manganelli, Rosa Iodice

**Affiliations:** ^1^ Department of Neurosciences, Reproductive and Odontostomatological Sciences University Federico II of Naples Naples Italy; ^2^ Department of Advanced Biomedical Sciences University of Naples Federico II Naples Italy; ^3^ Molecular Medicine IRCCS Fondazione Stella Maris Calambrone Pisa Italy; ^4^ IRCCS SDN (Istituto di Ricovero e Cura a Carattere Scientifico) Naples Italy

## Abstract

We describe an Italian family with adult‐onset pure hereditary spastic paraplegia due to biallelic variants in *POLR3A* gene [c.1909 + 22G > A and c.3839dupT (p.M1280fs*20]. MRI showed a mild hyperintensity of superior cerebellar peduncles and cervical spinal cord atrophy. The neurophysiological metrics about intracortical excitability showed higher values of motor thresholds and a significant reduction of short interval intracortical inhibition (SICI) in the patient with a more severe phenotype. Our multimodal evaluation further expands the wide phenotypic spectrum associated with mutations in the *POLR3A* gene. An extensive genotype–phenotype correlation study is necessary to explain the role of the many new mutations on the function of protein.

## Introduction

*POLR3A* gene encodes for the largest subunit of RNA polymerase III, an enzyme involved in the synthesis of several forms of RNA, including ribosomal RNA (rRNA) and transfer RNA (tRNA). Mutations *POLR3A* were first linked to young onset of hypomyelination, hypodontia and hypogonadotropic hypogonadism (4H leukodystrophy).^1^, ^2^, ^3^ Age of onset is typically in early childhood but later onset cases have also been reported.^4^, ^5^, ^6^ In the recent years, a wide neurological spectrum is reported in adult patients with *POLR3A* mutations without hypomyelination. Biallelic *POLR3A* variants have been frequently associated with hereditary spastic ataxia.^4^, ^5^, ^6^ The brain and spinal cord MRI images of these patients showed the hypoplastic corpus callosum, bilateral hyperintensity along the entire superior cerebellar peduncles, and the atrophy of the cervical spinal cord.^4^ Even more recently, Harting and coworkers described a cohort with basal ganglia involvement and extrapyramidal disorders such as parkinsonism and dystonia.^7^ Our multimodal evaluation further expands the phenotypic spectrum associated with mutations in the *POLR3A* gene, in fact, for the first time, we describe an Italian family with a pure hereditary spastic paraplegia (SPG) and biallelic *POLR3A* variants. In addition, in line with previous transcranial magnetic stimulation (TMS) studies performed on SPG patients,^8^ we carried out an extensive TMS battery to evaluate the function of intracortical circuits in the primary motor cortex.^9^

## Patients and Methods

### Subjects

The study was conducted according to the Declaration of Helsinki and approved by the Ethical Committee of University Federico II of Naples. Written informed consent was obtained from the patients. Clinical data including medical history, physical examination, neuropsychological battery, and magnetic resonance imaging scanning result from the patient were collected and analyzed.

### Neurophysiological examination

An extensive neurophysiological study was carried out with needle EMG, nerve conduction, visual evoked potentials (VEPs), brainstem auditory evoked potential (BAEPs), motor evoked potential (MEPs), somatosensory evoked potentials (SEPs).^10^, ^11^ We also carried out a systematic evaluation of intracortical circuits with resting motor threshold (RMT), active motor threshold (AMT), short interval intracortical inhibition (SICI), intracortical facilitation (ICF), long interval intracortical inhibition (LICI).^12^ Results from the patient were collected and analyzed.

### Next generation sequencing study

Genomic DNA was purified from blood sample using the standard phenol extraction protocol. The NGS study was performed as described by Dosi et al.^13^ using a customized bioinformatic pipeline^14^ to confirm the impact of mutations in silico according to ACMG guidelines.^15^

## Results

### Clinical findings

**Figure 1:**
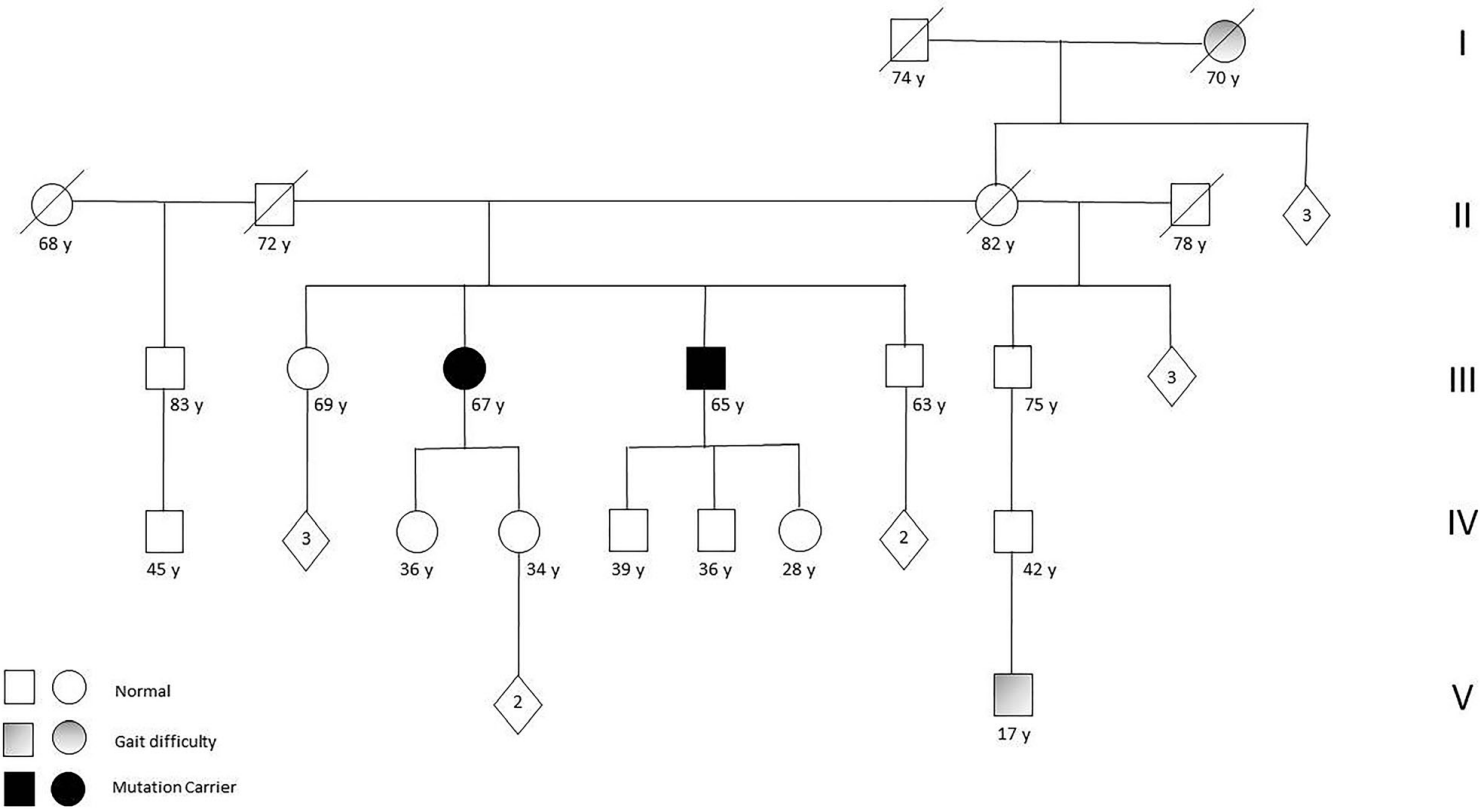
Complete pedigree of family with compound heterozygous mutations [c.1909 + 22G > A + c.3839dupT (p.M1280fs*20)] of *POLR3A* gene. Squares represent males; circles represent females; blank symbols represent normal subjects; gray filled symbols indicate patients with gait problems; black filled symbols represent symptomatic mutation carriers.

**Figure 2:**
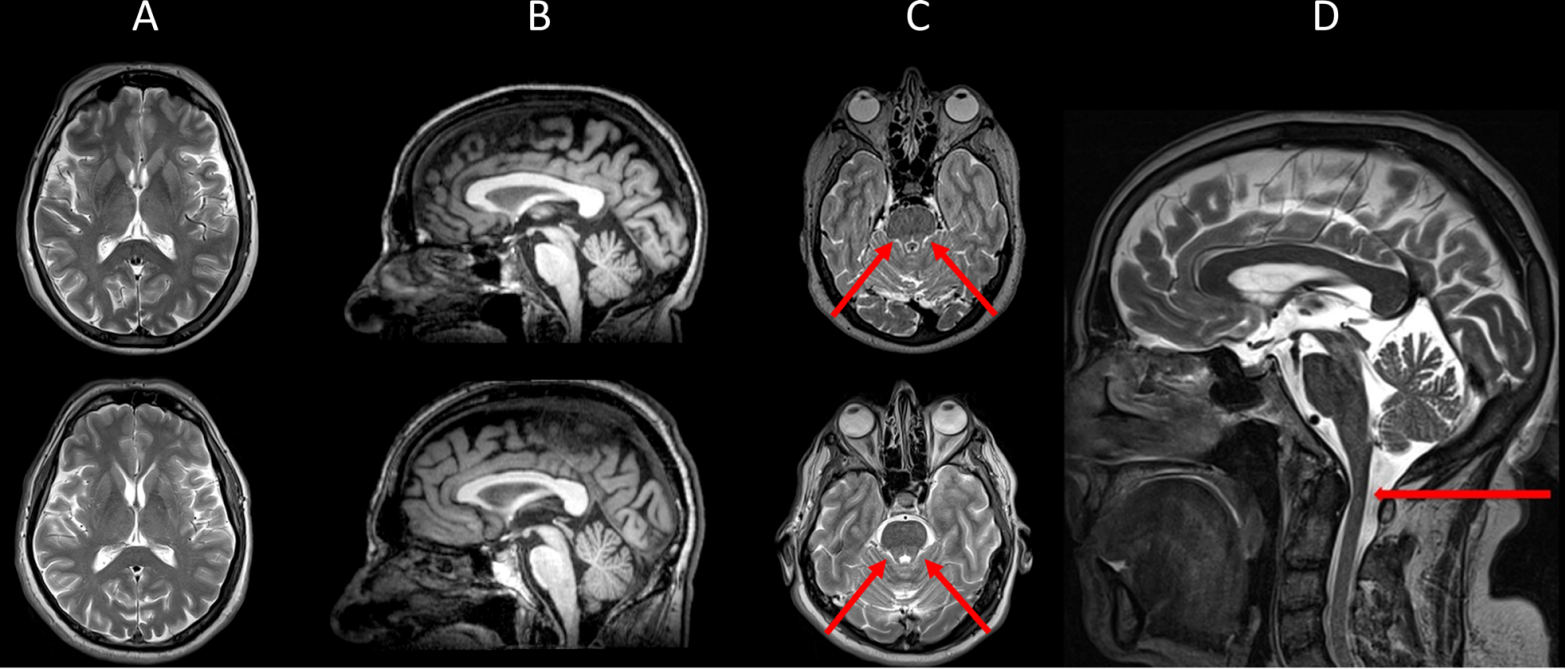
Brain MRI examination showing findings in patient III3 (upper row) and patient III 4 (lower row). Representative axial T2‐weighted (A, C) and reformatted sagittal T1‐weighted (B) images show absence of supratentorial atrophy or signal alterations (A, B). T2‐weighted images showing mild hyperintensity affecting both superior cerebellar peduncles (red arrows in C) and a slight spinal cord cervical atrophy (red arrows in D).

We reported two siblings from a southern Italian family: a 66‐year‐old female (III3, Fig. 1) and a 64‐year‐old male (III4, Fig. 1). They underwent first neurological examination at the age of 40 and 42 due to gait problems and received a clinical diagnosis of recessive pure spastic paraparesis. Their parents were not consanguineous, and a complete pedigree is shown in Figure 1. Both patients referred the symptoms’ onset in the second decade of life, characterized by a progressive and very slow gait disturbance. At the last neurological examination patient III3 had a score of 39 calculated with the use of spastic paraplegia rating scale (SPRS),^16^ while patient III4 had a score of 28. Both patients had a paraplegic spastic gait, although patient III3 used a bilateral support for walking. Increased muscle tone at lower limb with Babinski sign, despite normal tendon reflex, was noted. There were no signs of cerebellar or extrapyramidal involvement. Vibration was reduced at distal lower limbs. A detailed clinical evaluation is summarized in Table 1. The neuropsychological battery showed a short memory deficit. At the brain MRI (Fig. 2), both patients showed preserved cerebral and cerebellar volumes, without any supratentorial MRI signal changes. In both patients, a mild T2‐weighted hyperintensity was found, affecting both superior cerebellar peduncles and not associated to a significant volume loss. Finally, patient III4 underwent a cervical spinal cord MRI, that showed a mild volume loss without any signal alterations. The III3 patient refused to undergo a cervical spine examination.

**Table 1:** General findings.

	P III3	P III4
Sex	F	M
Age at onset	30	20
Age at exam	66	64
Cognitive deficits	+	+
Pyramidal system		
UL/LL spasticity	−/+	−/+
UL/LL tendon reflexes	N	N
Babinski	+	+
Peripheral motor system		
UL/LL weakness	N	N
Muscle atrophy	−	−
Cerebellar system		
Saccadic pursuit	−	−
Dysartria	−	−
Tremor	−	−
Ataxia	−	−
Vibration/surface sensitive	D	D
Urinary/fecal urgency	N	N
Dentation abnormalities	−	−
SPRS score	39	28
Neurophysiology		
Abnormal nerve conduction	−	−
Abnormal MEPs	+	+
Abnormal SEPs	+	+
Abnormal VEPs	+	+
Abnormal BAEPs	+	+
MRI		
Spinal cord atrophy	n.d	+
Cerebellar atrophy	−	−
TCC	−	−
SCP hyperintensity (FLAIR)	+	+
Hypomyelination	−	−

BAEPs, Brainstem Auditory Evoked Potential; D, decrease of clinical sign; I, increase of clinical sign; F, female; LL, lower limb; M, male; MEP, motor evoked potential; n.d., not done; N, normal/unchanged findings; SCP, superior cerebellar peduncle; SEP, cortical latencies of somatosensory evoked potentials; SPRS: spastic paraplegia rating scale; TCC, thin corpus callosum; UL, upper limb; VEP, cortical latencies of visual evoked potentials. +, clinical sign is present; −, clinical sign is absent.

### Neurophysiological and genetics findings

**Figure 3:**
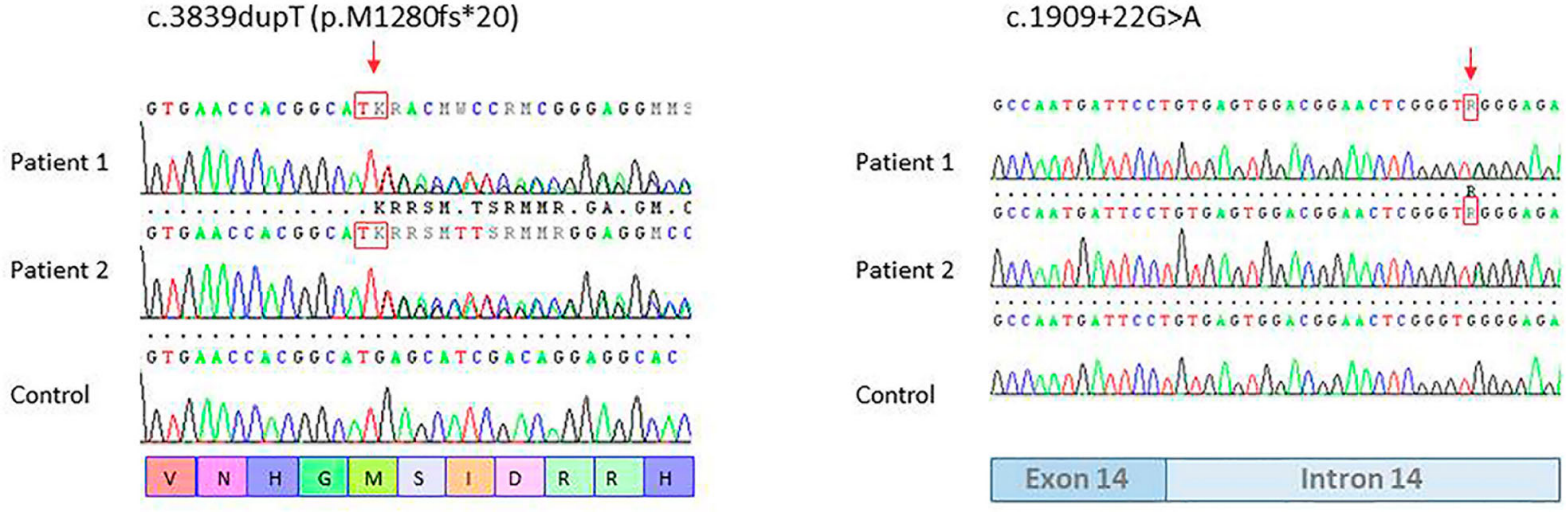
Electropherograms of the regions flanking the variants c.3839dupT (p.M1280Fs*20) and c.1909 + 22G > A of *POLR3A* gene in genomic DNA of patient III3, patient III4, and control.

Needle EMG, sensory, and motor nerve conduction studies were normal. SEPs following median and tibial nerve stimulation were abnormal in both patients along the central pathway for upper and lower limbs. VEPs revealed a damage of visual pathway and BAEPs showed the involvement of auditory pathway within the brainstem (Table 1). TMS showed a prolonged central motor conduction time (CMCT) for upper and lower limbs. In addition, TMS metrics of intracortical excitability showed in our patients an overall reduction of motor cortex excitability as demonstrated by higher values of motor thresholds (RMT and AMT). In addition, in patient III3 who showed a more severe phenotype, our data disclosed a significant reduction of SICI reflecting an impairment of intracortical GABAA circuits and higher motor thresholds respect to patient III4. Lastly, normal findings were obtained for ICF and LICI that are supposed to be underpinned by glutamatergic and GABAB circuits, respectively (Table 2). We identified biallelic variants in *POLR3A*, the described c.1909 + 22G > A, and the new c.3839dupT (p.M1280fs*20) (Fig. 3). The former is a relatively common disease allele in *POLR3A* found in compound heterozygosity in about 80% of the mutated patients.^4^ The latter gene variant affects an amino acid (Met1280) highly conserved across species and it predicts a frameshift with early protein termination.

**Table 2:** Cortical Excitability metrics.

	P III4	P III3	Normal values
RTM (%)	**55**	**70**	28–52
AMT (%)	**46**	**55**	21–39
SICI‐2 (%)	43.37	**85.65**	<63
SICI‐3 (%)	49.26	**81.82**	<70
ICF‐10 (%)	112.00	106.70	<194
ICF‐15 (%)	156.11	124.40	<232
LICI‐150 msec (%)	44.88	28.74	<64
LICI‐200 msec (%)	13.78	41.94	<59

Motor thresholds (% of maximum stimulator output), short interval intracortical inhibition (% of test MEP) at 2 and 3 msec interstimulus interval (ISIs), intracortical facilitation (% of test MEP) at 10 and 15 msec ISIs, long interval intracortical inhibition at 150 and 200 msec ISIs short‐latency afferent inhibition (% of test MEP) at +2, +4, +6, +8 msec after N20 latency.

Normal values are reported as mean + or ‐ 2 SD of control values, increased values in bold.

RMT, resting motor threshold; AMT, active motor threshold; SICI, short interval intracortical inhibition, ICF, intracortical facilitation; LICI, long interval intracortical inhibition.

## Discussion

Early reports of the clinical phenotype due to rare mutations of the *POLR3A* gene appeared quite homogeneous and characterized by infantile or young onset of a severe neurological syndrome with a diffuse brain MRI hypomyelination. Many of these patients also showed a non neurological involvement. Afterward, Minnerop et al. have stressed that the mutations of *POLR3A* gene are not so rare and a high proportion of patients with ataxia‐spastic syndrome showed a biallelic variants of *POLR3A* gene.^4^ In addition, the authors expanded the clinical spectrum. Specifically, the clinical onset was variable ranging from infantile to adulthood form, and at patients’ brain MRI there was no diffuse hypomyelination but bilateral hyperintense signals along the superior cerebellar peduncles, associated with cerebellar and spinal cord atrophy. Another recent paper reports an extrapyramidal syndrome with dystonia.^7^ Interestingly, most of the patients described so far, carried the same intronic mutation (c.1909 + 22G > A). The intronic variant c.1909 + 22G > A is present in almost all the populations included in gnomAD and it has an ultrarare allele frequency (MAF 0.001365). The base change is considered a relatively common mutant allele in *POLR3A*‐related phenotypes. In a large cohort of patients with a diagnosis of HSP or cerebellar ataxia of unknown origin mutations in *POLR3A* were identified in ~3.1% of cases, 80% of which were compound heterozygous for the c.1909 + 22G > A change.^4^

**Figure 4:**
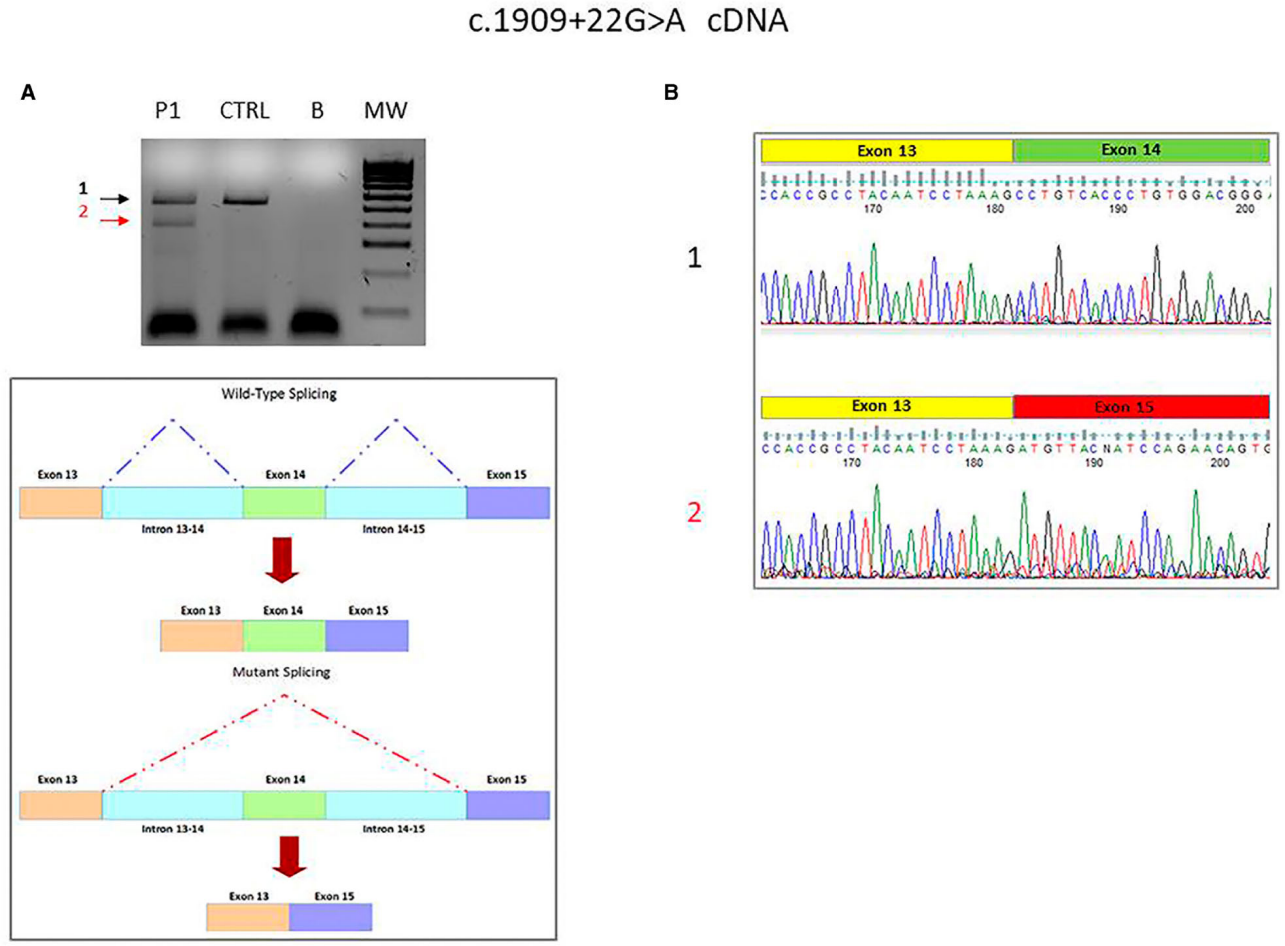
(A) Agarose gel electrophoresis of reverse transcription polymerase chain reaction products of the exonsr egion flanking the variant c.1909 + 22G > A of *POLR3A* transcript. Lane 1, P, patient; lane 2, CTRL, normal control; lane 3, B, blank; lane 4, MW, molecular weight DNA 100 bp ladder. (B) Electropherograms of reverse transcription polymerase chain reaction products of the exons' region flanking the variant c.1909 + 22G > A of *POLR3A* transcript in patient III3. 1: transcript amplification of the region encompassing exons 13 to 14 of *POLR3A* transcript in patient similar to normal control; 2: transcript amplification showing exon 14 skipping due to intronic retention or RNA instability in patient.

We reported the first Italian family with biallelic mutation of *POLR3A* gene and our data confirm many of controversial aspects analyzed previously. From the genetic point of view, our patients carried the common intronic mutation (c.1909 + 22G > A), so probably this mutation is present also in Italian population (Fig. 4). In addition, as frequently described by other authors the second variant is a new mutation that for the first time is a duplication. About the clinical aspect, the neurological presentation of our patients is peculiar, since it differs from classic leukodystrophy, hereditary spastic ataxia, or extrapyramidal syndrome associated with *POLR3A* gene mutations. In fact, our patients have a pure pyramidal involvement with spastic paraparesis gait. Accordingly, their brain MRI is substantially spared, except for a mild cervical spinal cord atrophy and hyperintensity of superior cerebellar peduncles. Moreover, the systematic evaluation of intracortical circuits suggests an impairment of intracortical excitability, and such alterations paralleled clinical phenotype. To our best knowledge, this is the first time that these circuits are analyzed in carriers of mutations of *POLR3A* gene.

In conclusion, our data suggest that mutations of the *POLR3A* gene should be also considered among the cause of pure hereditary spastic paraparesis, further expanding the spectrum of phenotypes associated with mutations of this gene. To explain this clinical heterogeneity, it would be necessary an extensive genotype–phenotype correlation study to clarify the role of the many new mutations found in association with the common mutation c.1909 + 22G > A.^17^ The study of intracortical circuits could give further information for the characterization of phenotype and could provide some pathophysiological insights about the function of POLR3A protein. We cannot draw conclusions since we studied only two patients but this could be the starting point for future studies.

## Conflict of Interest

The authors report no competing interests.

## Author Contributions

LR, FMS, RI designed the study and wrote the article. LR, AI, AF, LI collected and organized data. RD performed neurophysiological study, SC, GP, and AB performed neuroradiological study, RT, MB, and FMS performed genetic testing. LR, RD, SC, RT, AI, and GP prepared figures and tables. LR, FM, FMS, and RI revised the paper and made critical suggestions.
